# Liver Hematoma in Pregnancy: Challenges, Management Strategies, and Outcomes

**DOI:** 10.1097/og9.0000000000000070

**Published:** 2025-03-06

**Authors:** Antonio F. Saad, Khalil M. Chahine, George R. Saade, Baha M. Sibai

**Affiliations:** Division of Maternal-Fetal Medicine, Department of Obstetrics and Gynecology, Inova Fairfax Hospital, Falls Church, and the Division of Maternal-Fetal Medicine, Department of Obstetrics and Gynecology, Eastern Virginia Medical School, Norfolk, Virginia; and the Division of Maternal-Fetal Medicine, Department of Obstetrics and Gynecology, University of Texas Houston, Houston, Texas.

## Abstract

Liver hematoma is a rare but devastating complication of pregnancy, associated mainly with hypertensive disorders of pregnancy. Subcapsular liver hematomas complicate between 1 in 40,000 and 1 in 250,000 pregnancies. The risk is significantly higher in women with HELLP (hemolysis, elevated liver enzymes, and low platelet count) syndrome, with an incidence of 0.9–1.6% in this subgroup. Clinically, the condition arises as a result of vasospasm in the hepatic microcirculation, triggered by an imbalance in angiogenic factors such as soluble fms-like tyrosine kinase-1 and vascular endothelial growth factor, leading to hepatic ischemia, infarction, and subsequent hematoma formation. The right hepatic lobe is most frequently affected, and symptoms are often vague and nonspecific, necessitating a high index of suspicion for diagnosis. Early diagnosis can be made through imaging with ultrasonography or computed tomography. Patients with liver hematomas need highly individualized care. Optimal management remains unknown; historically, cases were managed surgically, but more recent case series have demonstrated that close observation with blood product replacement and surveillance of the hematoma with imaging is also a reasonable approach for stable patients. Although there is potential for liver recovery in most cases; severe cases can result in irreversible damage and complications such as hepatic rupture and death.

## Abstract 2

Liver hematoma in pregnancy, most commonly seen as a severe manifestation of hypertensive disorders of pregnancy, necessitates early diagnosis with advanced imaging techniques and individualized management.

The occurrence of a subcapsular hematoma is an uncommon yet potentially fatal outcome of preeclampsia, affecting between 1 in 40,000 and 1 in 250,000 pregnancies.^1^ Although rare, its high associated morbidity and mortality underscore the need for timely diagnosis and effective management strategies. This condition is linked predominantly to liver subcapsular vascular injury, leading to bleeding and hematoma formation, and it complicates about 0.9–1.6% of cases of HELLP (hemolysis, elevated liver enzymes, and low platelet count) syndrome.^1^ A severe variant of preeclampsia, HELLP syndrome is characterized by systemic endothelial dysfunction, coagulopathy, and microvascular damage, making it a major risk factor for subcapsular liver hematoma. Additional risk factors for subcapsular liver hematomas include advanced maternal age, multiparity, and obesity.^2^

A subcapsular liver hematoma frequently involves the right hepatic lobe. Its symptoms such as right upper-quadrant pain, shoulder pain, nausea, and hypotension are often vague and nonspecific, making early diagnosis challenging. Optimal management remains unknown; historically, cases were managed surgically, but more recent case series have demonstrated that close observation with blood product replacement is also a reasonable approach for stable patients.

Maternal mortality rates vary widely, depending on the timeliness of diagnosis and the management approach. In a recently published review that included 73 cases of liver hematoma in pregnancy from 2010 to 2019,^3^ maternal mortality decreased from 39% before 2003^4^ to around 15%^3^ in 2019; perinatal mortality rates showed only slight improvement.^3,4^

Despite advances in imaging and critical care, significant gaps remain in the understanding of the predictors of progression, long-term outcomes, and best practices for multidisciplinary care. This review aims to address key knowledge gaps, to provide updated recommendations in the form of a clinical practice algorithm, and to highlight areas for future research to improve maternal and fetal outcomes.

## PATHOPHYSIOLOGY OF HELLP SYNDROME

A severe variant of preeclampsia, HELLP syndrome is characterized by hemolysis, elevated liver enzymes, and low platelet count.^5^ The pathophysiology of subcapsular liver hematoma is believed to be attributable to vasospasm in the hepatic microcirculation,^6^ widespread endothelial dysfunction, and microvascular injury, triggered primarily by an imbalance between angiogenic and antiangiogenic factors such as vascular endothelial growth factor and soluble fms-like tyrosine kinase-1.^7,8^ These disturbances lead to systemic vasospasm, which reduces blood flow to the liver and other organs, resulting in ischemia and subsequent hepatocellular damage and potential bleeding complications.^9^ If this process is severe and prolonged, it may cause large hepatic infarcts and progress to microvascular bleeding, subcapsular hematoma formation, and hepatic rupture.

Histopathologic findings often reveal periportal hemorrhage, fibrin deposition, and microvesicular fatty infiltration within hepatocytes, indicative of ongoing necrosis and inflammation.^10^ Biopsies of the liver in patients with HELLP syndrome consistently show these hallmark pathologic changes, correlating with the clinical severity of the syndrome. Despite advancements, laboratory markers often poorly correlate with the severity of histologic damage.^10^

## CLINICAL PRESENTATION AND DIAGNOSIS

An unruptured liver hematoma represents a contained collection of blood within the liver tissue. Liver subcapsular hematoma can manifest through a range of symptoms, all of which are nonspecific (Table 1). These symptoms result from stretching of the Glisson capsule as underlying blood accumulates. This condition may lead to liver rupture or infarction, posing severe risks to both the mother and the fetus if not quickly identified and treated.

**Table 1.** Clinical Presentation of HELLP Syndrome Compared With Subcapsular Liver Hematoma

Clinical Presentation of HELLP	Clinical Presentation of Subcapsular Liver Hematoma
Epigastric or right upper-quadrant pain Nausea or vomiting Viral-like syndrome symptoms (eg, malaise) Hypertension Petechial hemorrhages or bruises	Epigastric pain or right upper-quadrant pain Shoulder or neck pain Abdominal distention and hemoperitoneum Pleural effusion Shock (eg, hypotension, tachycardia) Relapsing hypotension Sudden change in fetal heart rate tracing

HELLP, hemolysis, elevated liver enzymes, and low platelet count.

Ruptured liver hematoma escalates quickly to a critical condition. With liver rupture, abdominal distention secondary to hemoperitoneum can occur, along with symptoms of hypovolemia and shock. Such presentations might be misdiagnosed as pulmonary embolism, particularly if accompanied by right-sided pleural effusion, or may be mistaken for other hepatobiliary conditions such as acute cholecystitis or pancreatitis. Imaging is recommended for any patient with HELLP syndrome experiencing any abdominal or peritoneal symptoms such as severe epigastric pain in association with shoulder pain or signs of sudden acute hypovolemia such as tachycardia or hypotension regardless of severity of transaminitis because these tests often fail to align with abnormalities detected on liver imaging.^10^

**Fig. 1.**
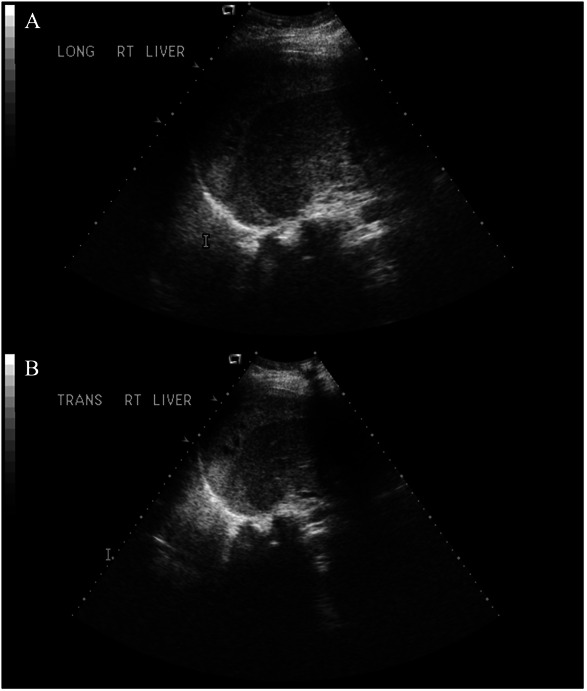
Longitudinal view (**A**) and transverse (**B**) ultrasound imaging of the liver of a patient with subcapsular hematoma.

**Fig. 2.**
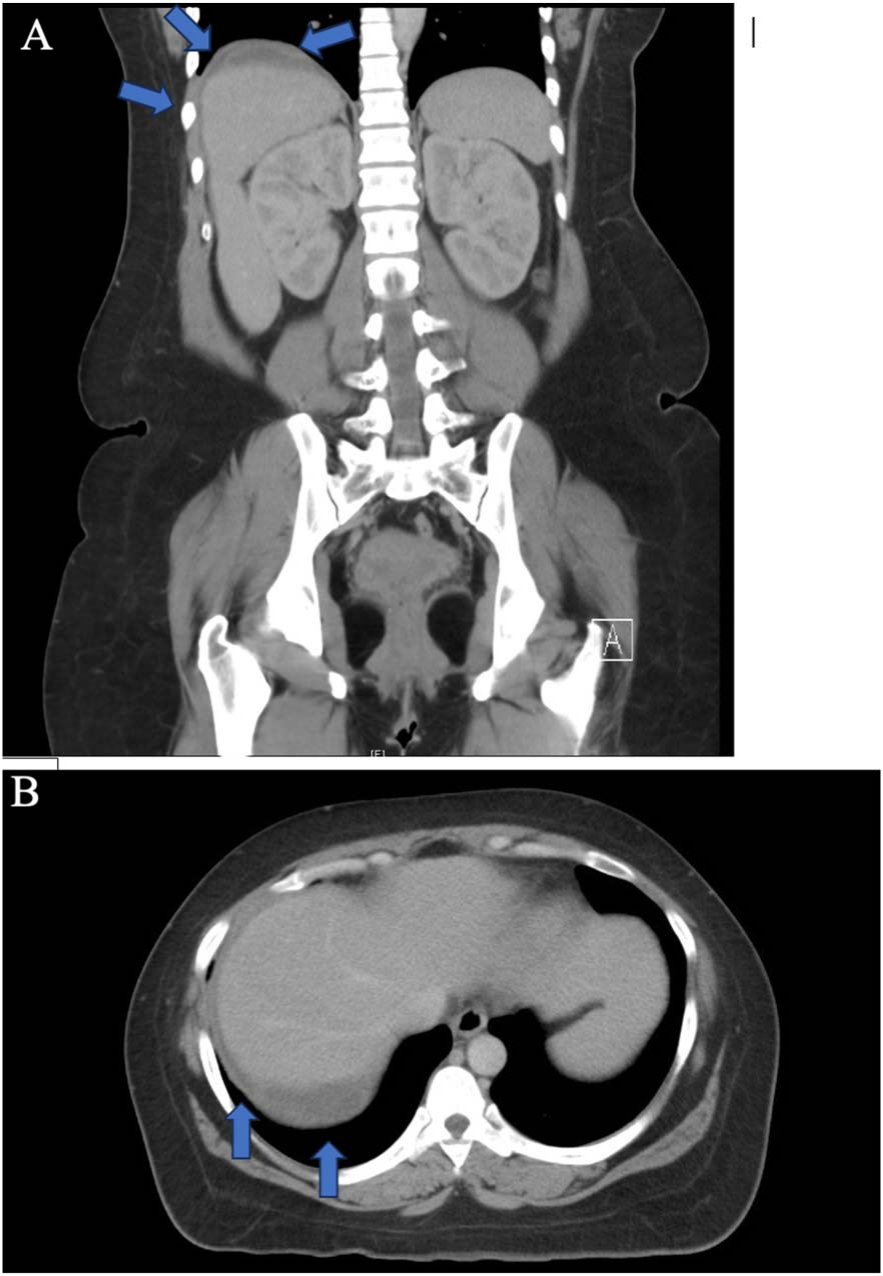
Frontal (**A**) and transverse (**B**) planes of computed tomography imaging of the liver of a patient with subcapsular hematoma (*arrows*).

Quick and accurate diagnosis of liver hematoma and rupture is essential. Ultrasonography is an effective initial diagnostic tool that can be used at the bedside (Fig. 1). However, it may not detect hematomas in the posterior lobe of the liver. Both computed tomography (CT) and magnetic resonance imaging are highly sensitive in detecting liver rupture and assessing the size and extent of the hematoma.^11,12^ However, CT (Fig. 2) is often preferred because of its greater accessibility, speed, and suitability for potentially unstable patients.

*Saad. Liver Hematoma in Pregnancy. O&G Open 2025*.

*Saad. Liver Hematoma in Pregnancy. O&G Open 2025*.

## EVOLVING STRATEGIES IN THE MANAGEMENT OF LIVER HEMATOMA IN PREGNANCY: FROM SURGICAL INTERVENTIONS TO MINIMALLY INVASIVE TREATMENTS

Historically, surgery has been the primary approach for treating subcapsular hematoma.^13^ Despite a lack of definitive evidence guiding clinical decisions, medical literature reports successful outcomes with alternative treatment methods^14^ such as minimally invasive transcatheter hepatic artery embolization.^3^

A recent published review described the management of 73 reported cases of liver hematoma in pregnancy from 2010 to 2019, which was categorized into four approaches: 1) surgical, 2) conservative, 3) embolization with or without surgery, and 4) liver transplantation.^3^ Surgical interventions often begin with a laparotomy, followed by evacuation or drainage of the hematoma or the peritoneal cavity, abdominal packing, or use of collagen sponges and liver sutures to provide hemostasis. More complex procedures include flap formation, partial liver resection, and hepatic artery ligation.^3^

Conservative treatments are focused on stabilizing the patient, including fluid resuscitation and other supportive measures, with serial ultrasound or CT scans to monitor the progress of the hematoma.^3^ A minimally invasive approach through hepatic artery embolization also showed promising fetal outcomes.^3^ Only one case of liver transplantation is reported, with both the mother and the child surviving.^15^

All approaches included transfusion of blood and blood products for management of blood loss and treatment of coagulopathies, administration of intravenous antibiotics to prevent or treat infections, and hemodialysis or hemofiltration in cases of compromised kidney function. These varied surgical and conservative approaches highlight the multifaceted and personalized medical strategies necessary to manage the complex and potentially life-threatening situation of liver hematoma in pregnancy. Surgery alone, which is more common in underresourced nations, led to the poorest fetal outcomes, often in the context of late presentation and lack of antenatal care.^3^

## CLINICAL MANAGEMENT

**Fig. 3.**
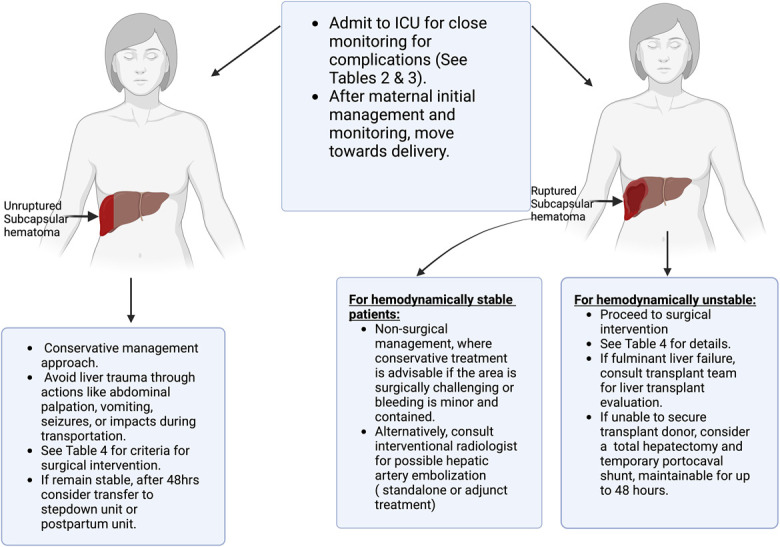
Summary of the main steps for management. ICU, intensive care unit. Created in BioRender. https://BioRender.com/t28y094.

Figure 3 summarizes a practical approach to clinical management. Initial treatment for patients with liver hematoma involves admission to an intensive care unit for close monitoring and early detection of signs of rupture (Tables 2 and 3). It is imperative to be prepared for the possible need for massive transfusion to counteract any hemodynamic compromise or coagulopathy. In cases involving HELLP syndrome or preeclampsia with severe features, treatment should include magnesium sulfate for eclampsia prevention and prompt antihypertensive therapy in cases of severe hypertension. Fetal monitoring is indicated if the patient is still pregnant and at a gestational age beyond which neonatal resuscitation would be offered at the institution. The decision to deliver should prioritize maternal well-being, with delivery for fetal indications deferred until maternal condition can be stabilized. As soon as the maternal condition is stabilized, arrangements for an urgent cesarean delivery can be initiated, balancing the risks and benefits as in other emergent scenarios.

*Saad. Liver Hematoma in Pregnancy. O&G Open 2025*.

**Table 2.** Maternal Complications of Subcapsular Liver Hematoma

Complication	Identification	Management
Rupture of hematoma	Sudden onset of severe abdominal pain, signs of hypovolemic shock (eg, tachycardia, hypotension), and abdominal distension	Immediate surgical intervention, blood transfusion, and hemodynamic stabilization
Hemorrhagic shock	Signs of shock (eg, rapid pulse, low blood pressure, and reduced urine output); laboratory findings of anemia and coagulopathy	Volume resuscitation with crystalloids or blood products and addressing the source of bleeding
Liver failure	Jaundice, coagulopathy, encephalopathy, and altered liver function tests	Supportive care, including management of encephalopathy and renal dysfunction; potential need for liver transplantation in severe cases
Disseminated intravascular coagulation	Prolonged clotting times, decreased platelets, decreased fibrinogen, and elevated D-dimer	Treatment of underlying cause, transfusion of platelets or clotting factors, and supportive care
Infection	Fever, elevated white blood cell count, and potential presence of localized pain or systemic signs of infection	Antibiotic therapy and drainage of any abscesses if present
Acute kidney injury	Reduced urine output, elevated serum creatinine and urea, fluid overload, and electrolyte disturbances	Optimization of fluid status, avoidance of nephrotoxic drugs, and in severe cases, renal replacement therapy

**Table 3.** Intensive Care Unit Monitoring for the First 48 Hours

Monitoring Parameter	Criteria and Frequency
Hemodynamic status	Continuous BP, HR, and MAP monitoring (target MAP above 55 mm Hg); assess for signs of shock
Laboratory values	Serial monitoring every 6–12 h: Hemoglobin and hematocrit (track trends for active bleeding) Coagulation panel (PT, aPTT, INR) Fibrinogen (target above 150 mg/dL) Platelet count ABGs
Skin perfusion	Assess for skin mottling or delayed capillary refill (indicators of poor perfusion)
Ultrasound imaging	Perform bedside ultrasonogram every 48 h or immediately if hemodynamic or laboratory abnormalities occur
CT scan	If clinical suspicion remains and negative ultrasound finding or worsening clinical status

BP, blood pressure; HR, heart rate; MAP, mean arterial pressure; PT, prothrombin time; aPTT, activated partial thromboplastin time; INR, international normalized ratio; ABG, arterial blood gas; CT, computed tomography.

For the first 48 hours, close monitoring of patients with subcapsular hematoma in the ICU is essential. This table outlines the parameters to be monitored closely, along with recommended frequency, to detect any signs of deterioration without unnecessary movement or imaging.

### Unruptured Liver Hematoma

In cases in which a patient has an unruptured subcapsular liver hematoma and is hemodynamically stable, a conservative management strategy is recommended (Fig. 3).^13,16,17^ This approach helps prevent additional trauma to the liver. It is crucial to monitor maternal status and to address any coagulation abnormalities closely. To prevent any risk of rupturing the hematoma, it is imperative to avoid anything that may cause rupture of the hematoma, including palpation of the upper abdomen or liver, vomiting, seizures, or any abdominal impact that could occur during patient transport, because these actions can lead to a sudden increase in intra-abdominal pressure.^12,18^ If conservative management is used, serial imaging tests such as ultrasound or CT scans are advised to assess the evolution of the hematoma and the presence of intraperitoneal bleeding. The frequency of serial imaging depends on the maternal hemodynamic status and coagulation changes.

Surgical intervention is indicated in cases of resistant coagulopathy such as clinical disseminated intravascular coagulation unresponsive to transfusions, characterized by prolonged clotting times and low fibrinogen levels. Persistent hemodynamic shock despite adequate resuscitation, including prolonged vasopressor use or continued transfusion needs attributable to failed initial resuscitative efforts, also warrants surgical consideration. Table 4 gives criteria indicative of clinical instability necessitating operative management.

**Table 4.** Criteria for Surgical Intervention

Criteria	Description and Threshold
Development of coagulopathy	Development of resistant coagulopathy, specifically clinical disseminated intravascular coagulation unresponsive to multiple blood components Includes criteria such as prolonged PT (more than 15 seconds), INR more than 1.5–2.0, fibrinogen less than 150 mg/dL, and aPTT more than 45 s
Hemodynamic shock despite resuscitation	*Persistent shock* despite adequate resuscitation (defined as SBP below 90 mm Hg or MAP below 55 mm Hg and heart rate above 110 bpm) If prolonged vasopressor use is required, surgery is indicated.
Failed transfusion response	Continued need for transfusion despite blood products, indicating failure of initial resuscitative efforts (eg, fluids, initial 1–2 L transfusion) excluding other causes of shock

PT, prothrombin time; INR, international normalized ratio; aPTT, activated partial thromboplastin time; SBP, systolic blood pressure; MAP, mean arterial pressure.

Complete resolution of the hematoma may take several months.^12^ Outpatient follow-up may include additional imaging to monitor for hematoma resolution, serial laboratory tests to assess liver function and coagulation status, and close clinical surveillance for any signs of deterioration. Patients should be discharged only with clear stability, no ongoing bleeding, and access to prompt medical care if complications arise.

### Ruptured Liver Hematoma

**Fig. 4.**
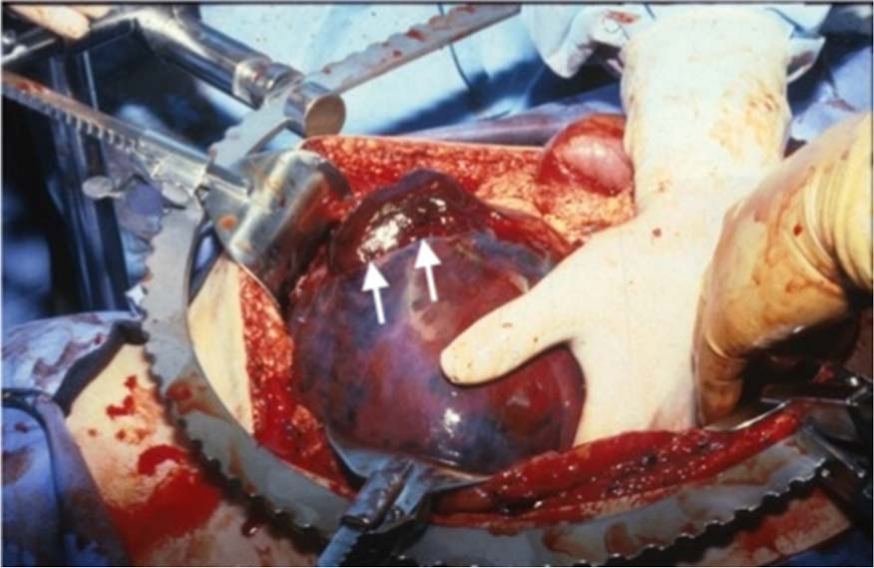
Intraoperative findings showing site of rupture of a large subcapsular liver hematoma. *White arrows* point to the site of rupture. Image courtesy of Dr. Baha Sibai. Used with permission.

When a liver rupture is suspected (Fig. 3), it is essential to consider surgical intervention and to seek the expertise of a team skilled in liver surgery. At laparotomy (Fig. 4), many surgical methods are available, depending on the extent of bleeding. These include abdominal packing, drainage, vascular ligation of the portal vein or hepatic artery branches, application of an omental patch, or a partial liver resection.^4,19–21^

*Saad. Liver Hematoma in Pregnancy. O&G Open 2025*.

In situations in which there is unmanageable liver bleeding resulting from persistent disseminated intravascular coagulation or rapidly worsening acute liver failure, a liver transplantation may be considered. According to our experience, the need for a liver transplantation is infrequent; early decisions regarding transplantation are essential to improve the chances of finding a suitable organ donor in time. If uncontrollable hemorrhage occurs and no organ donor is available, achieving hemodynamic stabilization through a total hepatectomy and the creation of a temporary portocaval shunt is a viable option. This anhepatic phase can be maintained for up to 48 hours before a transplantation is performed in a subsequent operation.^4,22^

For hemodynamically stable patients, nonsurgical management may be an option. Conservative treatment is advisable in scenarios in which the affected area is not easily accessible surgically or when the bleeding is minor, self-contained, and without associated hemodynamic compromise. Many cases have been reported in which hepatic ruptures linked to HELLP syndrome were successfully managed without surgery.^4,13,20,23^ Another nonsurgical alternative is hepatic artery embolization by an interventional radiologist. This technique can be effective as a stand-alone treatment or in conjunction with surgery.^3,24–26^

## SUBSEQUENT PREGNANCY

In managing future pregnancies, it is important to monitor for early indicators of preeclampsia and HELLP syndrome. The recurrence rate of subcapsular hematoma is currently unclear. Reports in the literature describe six subsequent pregnancies without complications after an initial hepatic hematoma, with four of these cases linked to preeclampsia or HELLP syndrome.^27–29^ One instance of recurrent liver hematoma has been documented: The initial event involved an unruptured hematoma managed conservatively, and the second, characterized by hematoma rupture, was managed with arterial embolization and surgical intervention. Imaging during the second event revealed multiple pseudoaneurysms at the bleeding site, and both episodes occurred postpartum.^28^ Among published case reports,^3,27–34^ 16 of 18 patients with subcapsular hematomas were identified as having HELLP syndrome during their initial pregnancies, and three of these cases were complicated by recurrent subcapsular liver hematoma.

In addition, in a recent case series,^30^ four individuals experienced a total of five subsequent pregnancies after their index pregnancies, during which all had HELLP syndrome. Among these, two individuals experienced recurrent HELLP syndrome, with one also developing recurrent subcapsular liver hematoma.

According to the U.S. Preventive Services Task Force,^34^ women with a history of preeclampsia, particularly with prior adverse events, should start low-dose aspirin as prevention. It is unclear whether this also helps prevent the risk of recurrent liver hematoma.

## CONCLUSION

The management of liver hematoma in pregnancy, particularly in the context of HELLP syndrome and preeclampsia, demands a comprehensive and careful approach. Recovery of the liver is generally anticipated, yet the possibility of extensive infarction exists, which can lead to hepatic rupture and, in severe cases, death. Treatment strategies, whether they lean toward surgical intervention or conservative management, hinge on the patient's stability and the specific characteristics of the hematoma. The key to successful management lies in vigilant monitoring and a readiness to swiftly address any arising complications, underscoring the need for individualized and responsive medical care in these complex and high-risk situations. However, it is important to acknowledge the limits of available data; much of the current understanding is derived from case reports and case series. This underscores the need for further research such as national registries to inform evidence-based practice and to refine management strategies.
